# A narrative review on yoga: a potential intervention for augmenting immunomodulation and mental health in COVID-19

**DOI:** 10.1186/s12906-022-03666-2

**Published:** 2022-07-18

**Authors:** Indranill Basu-Ray, Kashinath Metri, Dibbendhu Khanra, Rishab Revankar, Kavitha M. Chinnaiyan, Nagaratna Raghuram, Mahesh Chandra Mishra, Bhushan Patwardhan, Manjunath Sharma, Ishwar V. Basavaraddi, Akshay Anand, Shrinath Reddy, K. K. Deepak, Marian Levy, Sue Theus, Glenn N. Levine, Holger Cramer, Gregory L. Fricchione, Nagendra R. Hongasandra

**Affiliations:** 1 Cardiologist & Cardiac Electrophysiologist, Memphis VA Medical Center, 1030 Johnson Ave, Memphis, TN 38104 USA; 2 The University of Memphis, Memphis, TN USA; 3 All India Institute of Medical Sciences, Rishikesh, Uttarakhand India; 4 Department of Yoga, Central University of Rajasthan, Bandar Seendri, Rajasthan India; 5 New Cross Hospital, Heart and Lung Centre, Royal Wolverhampton NHS Trust, Wolverhampton, UK; 6 Icahn School of Medicine at Mount Sinai, New York, NY USA; 7 Department of Internal Medicine, Oakland University William Beaumont School of Medicine, Royal Oak, MI USA; 8 Swami Vivekananda Yoga Anusandhana Samsthana, Bangalore, Karnataka India; 9 Mahatma Gandhi University of Medical Sciences & Technology, Jaipur, Rajasthan India; 10 University Grants Commission, New Delhi, India; 11 Anveshana Research Laboratories, Swami Vivekananda Anusandhana Samsthana (SVYASA University), Bangalore, Karnataka India; 12 Morarji Desai National Institute of Yoga, Ministry of AYUSH, Govt. of India, New Delhi, India; 13 Department of Neurology, Post Graduate Institute of Medical Education and Research, Chandigarh, India; 14 Public Health Foundation of India, New Delhi, India; 15 Department of Physiology, All India Institute of Medical Sciences, New Delhi, India; 16 Cardiology Section, Baylor College of Medicine, Michael E. DeBakey VA Medical Center, Houston, TX USA; 17 Department of Internal and Integrative Medicine, University of Duisburg-Essen, Essen, Germany; 18 Department of Psychiatry, Benson-Henry Institute for Mind-Body Medicine, Massachusetts General Hospital, Boston, MA USA

**Keywords:** Catastrophization, Complementary therapies, Covid-19, Immunomodulation, Psychological stress, Yoga

## Abstract

**Background:**

The ongoing novel coronavirus disease 2019 (COVID-19) pandemic has a significant mortality rate of 3–5%. The principal causes of multiorgan failure and death are cytokine release syndrome and immune dysfunction. Stress, anxiety, and depression has been aggravated by the pandemic and its resultant restrictions in day-to-day life which may contribute to immune dysregulation. Thus, immunity strengthening and the prevention of cytokine release syndrome are important for preventing and minimizing mortality in COVID-19 patients. However, despite a few specific remedies that now exist for the SARS-CoV-2virus, the principal modes of prevention include vaccination, masking, and holistic healing methods, such as yoga. Currently, extensive research is being conducted to better understand the neuroendocrinoimmunological mechanisms by which yoga alleviates stress and inflammation. This review article explores the anti-inflammatory and immune-modulating potentials of yoga, along with its role in reducing risk for immune dysfunction and impaired mental health.

**Methods:**

We conducted this narrative review from published literature in MEDLINE, EMBASE, COCHRANE databases. Screening was performed for titles and abstracts by two independent review authors; potentially eligible citations were retrieved for full-text review. References of included articles and articles of major non-indexed peer reviewed journals were searched for relevance by two independent review authors. A third review author checked the excluded records. All disagreements were resolved through discussion amongst review authors or through adjudication by a fourth review author. Abstracts, editorials, conference proceedings and clinical trial registrations were excluded.

**Observations:**

Yoga is a nonpharmacological, cost-effective, and safe intervention associated with several health benefits. Originating in ancient India, this vast discipline consists of postures (asanas), breathing techniques (pranayama), meditation (dhyana/dharana), and relaxation. Studies have demonstrated yoga’s ability to bolster innate immunity and to inhibit cytokine release syndrome. As an intervention, yoga has been shown to improve mental health, as it alleviates anxiety, depression, and stress and enhances mindfulness, self-control, and self-regulation. Yoga has been correlated with numerous cardioprotective effects, which also may play a role in COVID-19 by preventing lung and cardiac injury.

**Conclusion and relevance:**

This review paves the path for further research on yoga as a potential intervention for enhancing innate immunity and mental health and thus its role in prevention and adjunctive treatment in COVID-19.

## Introduction

Coronavirus disease (COVID-19) is a highly contagious viral disease that has affected 238,349,712 people worldwide as of October 9, 2021. Its outbreak was initially reported in 2019 in Wuhan, Hubei Province, China. Nearly 5 million deaths had been reported worldwide as of the first week of October 2021. Many countries are still “locked down” to prevent extensive spread of infection, whereas others have relaxed these measures; even so, social isolation measures are still generally recommended, at least to some extent. Many argue that easing social restrictions has contributed to spikes in the number of cases nationwide [1–5].

Given the limited treatment options and the emergence of multiple strains with variable susceptibility to vaccines, clinicians are searching for other interventions to aid in the prevention and treatment of COVID-19. In the context of integrative medicine, yoga is a mind-body discipline that promotes healthy living through various components, such as the practice of postures (asana), breathing techniques (pranayama), concentration (dharana), and meditation (dhyana) [2, 6]. A growing body of evidence suggests that yoga practice leads to better integrative management of a number of non-communicable diseases that share the same pathophysiology, including cardiovascular diseases, stroke, and diabetes mellitus type II. The underlying reasoning is that these diseases, like COVID-19, express rogue immunological aberration, resulting in many of their manifestations, which are often triggered or exacerbated by stress [2, 7]. A meta-analysis of ten randomized controlled trials including 431 individuals suggested that yoga programs improved exercise capacity (mean change 2.69, 95% confidence interval 1.39- 3.99) and health related quality of life (mean change 1.24, 95% confidence interval − 0.37- 2.85) among patients with chronic ailments namely heart disease, chronic obstructive pulmonary disease and stroke when compared with normal care [8]. Consistent practice of yoga strengthens innate and adaptive immunity and helps to enhance physiological functions, such as respiration, digestion, circulation, and hormone production [2, 9–11].

In this review article, we discuss inflammatory, infectious, and psychosocial aspects of COVID-19 and explore the anti-inflammatory and immune-modulating potentials of yoga, along with its role in reducing risk factors for immune dysfunction and impaired mental health. We propose yoga as an intervention for expediting recovery in patients with COVID-19 and for enhancing innate immunity and mental health to bolster resistance to the virus [2].

## Methods

We conducted this narrative review from published literature in MEDLINE, EMBASE, and COCHRANE databases. Articles were retrieved from database searches using keywords related to complementary therapy, COVID-19, immunomodulation, psychological stress, and yoga. Observational and experimental studies and discussing the role of yoga in anxiety, immunomodulation, and COVID-19 were considered relevant for this narrative review. Screening was performed for titles and abstracts by two independent review authors; potentially eligible citations were retrieved for full-text review. References of included articles and articles of major non-indexed peer reviewed journals were searched for relevance by two independent review authors. A third review author checked the excluded records. All disagreements were resolved through discussion amongst review authors or through adjudication by a fourth review author. Abstracts, editorials, conference proceedings and clinical trial registrations were excluded. Only articles in English language were included.

## SARS-COV-2 infection

SARS-CoV-2, the coronavirus that causes COVID-19, is an acute infectious agent that enters the body through the respiratory system. Droplet transmission is understood to be the primary mode of transmission. Mounting evidence also suggests airborne transmission, although the World Health Organization has yet to confirm this. A person can become infected when his or her mucus membrane (within the nose, eyes, or mouth) comes into contact with the respiratory secretions of an actively infected person discharging virus particles. Having entered the body, the SARS-CoV-2 virus uses its S-spike to bind angiotensin-converting enzyme (ACE)-2 receptors as an entry point into the cell. The ACE2 receptor is expressed primarily in both type I and type II pneumocytes but also in other types of cells, including endothelial cells. Thus, it plays a vital role in vascular integrity and hemodynamic regulation [12–14].

Evidence indicates that cardiac involvement is ubiquitous in patients with COVID-19, particularly in hospitalized patients [14]. Patients with cardiac risk factors or established cardiovascular disease have heightened vulnerability, along with worse mortality and morbidity profiles. In various studies, nearly 30% of afflicted patients had hypertension and 15% had preexisting cardiovascular disease [15, 16].

## Role of immunity in COVID-19

The human immune system comprises multiple organs, such as the spleen, thymus, lymph nodes, tonsils, and bones. Immune cells and their products destroy the intruding infective organisms and neutralize them. The immune system includes both innate immunity and adaptive immunity. Innate immunity is the rapid-acting first line of defense that effectively inhibits infective agents from entering the body. However, if this line of defense fails, the immune system activates adaptive immunity, which is important to control most viral infections. The emerging picture reveals that CD4+ T cells, CD8+ T cells and neutralizing antibodies has important role in COVID-19 and thus its prevention and management [17].

Innate immunity is garnered to restrict infections by novel pathogens, such as SARS-CoV-2. This elaborate immunological cascade appropriately arrests the disease and helps to initiate the repair mechanism, thus ensuring satisfactory resolution of the infection and generating targeted resistance to defend the body against reinfection by the same organism [18]. The adaptive immune system involves T lymphocytes, B lymphocytes, and pathogen-specific antibodies in addition to the proinflammatory cytokines and chemokines that help to eliminate the pathogen [19]. Although these processes are very potent and effective, they can render bystander damage to the body’s own cells and organs.

**Fig. 1:**
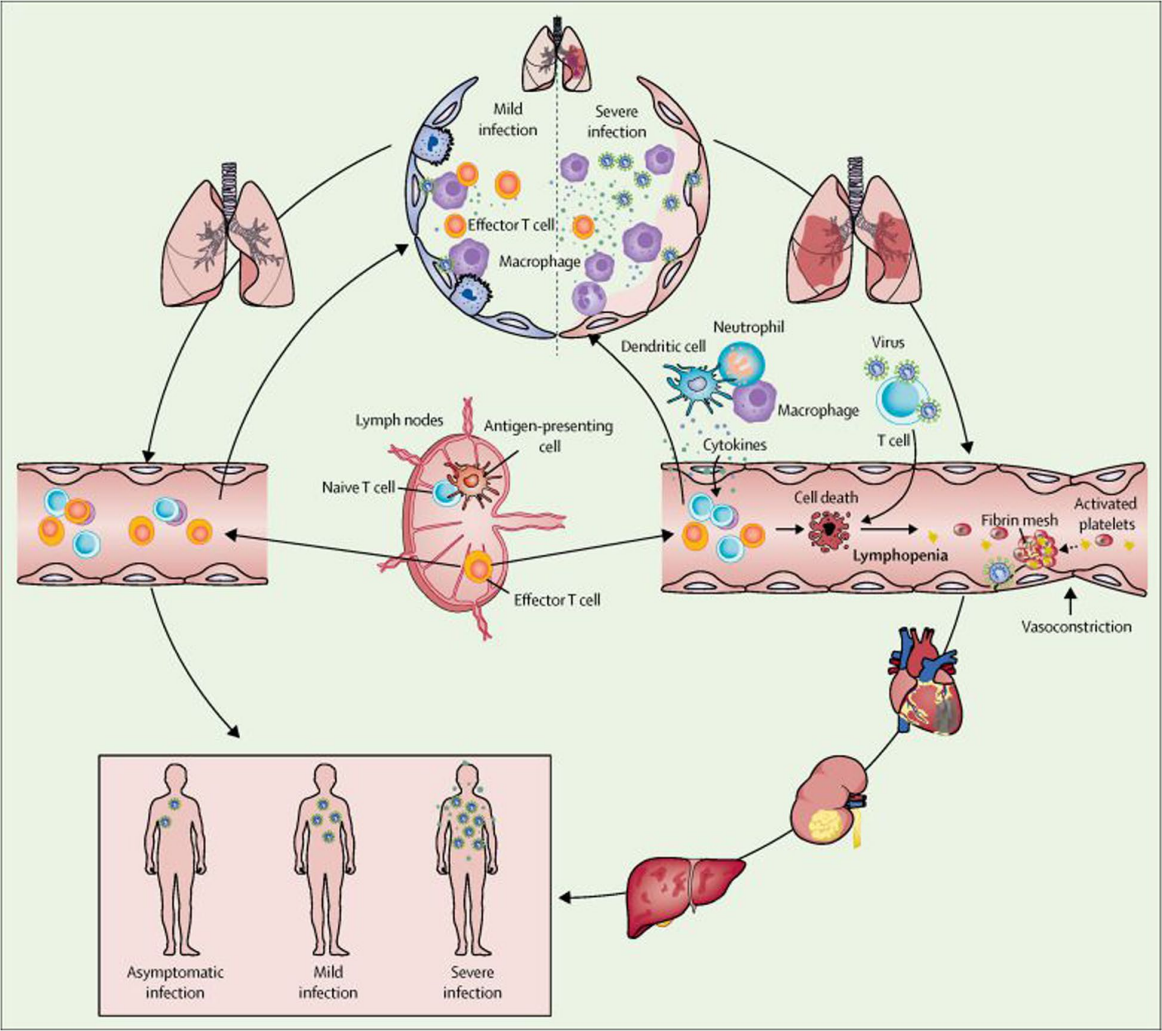
Pathological changes in lungs in early and severe stages of COVID-19 [From “SARS-CoV-2 and viral sepsis: observations and hypotheses” by Li H, Liu L, Zhang D, et al.; accessed 10 April 2021] [Permission for re-use granted by Elsevier COVID-19 resource center guidelines] [21]

Infection with COVID-19 presents with three different clinical scenarios: (1) asymptomatic carriers who have adequately functioning innate immunity; (2) symptomatic carriers with mild symptoms who achieve spontaneous recovery as their innate immunity detects infection and restricts it, while generating adaptive immunity that optimally gets rid of the virus; and (3) patients who develop moderate to severe illness and either recover or die from the infection [20]. In this third category of patients, the body’s immune system, in both its innate and adaptive expressions, is activated. In those who die, the immune system is overwhelmed, leading to cytokine release syndrome (CRS), a massive, cascading release of cytokines that initiates widespread destruction and multiorgan failure, ultimately leading to death [13]. In essence, the virus does not directly kill but instead initiates an immunological reaction that is morbid and occasionally fatal (Fig. 1). It is therefore unfortunate that the resources harnessed by the body to kill the virus largely outweigh the appropriate levels needed and instead produce tissue destruction, organ failure, and eventually death. Interleukin (IL)-6 is the primary candidate cytokine suspected of perpetrating this fatal reaction [14, 15]. This knowledge has spawned initiatives to block IL-6 using receptor inhibitors, including biologics like tocilizumab, which are undergoing trials in moderately to severely ill patients with COVID-19 [19].

An optimal innate immune response may thus play a vital role in the prevention and early disposal of most COVID-19 infections. A response of this nature is believed to occur in 80% or more of those infected, who either are asymptomatic or develop mild symptoms that defervesce and culminate in an uneventful recovery. The precise cause of immune dysfunction and CRS led by the overproduction of IL-6 is unknown. Nonetheless, considerable evidence points to the fact that the severity of the disease is based on the immune response to the virus, among other factors [22].

## Pandemics, immunity, and mental health

Remdesivir, the antiviral agent effective against COVID-19, only shortens the illness timetable by around 33% [23]. The antiviral treatments recently approved by the FDA would lead to resistance if randomly used. Moreover, their efficacy is not absolute and is only effective if started early in the course of the infection. These limitations render preventive measures—including vaccination, hygiene, social distancing, and personal protective equipment—to be the primary means of managing the COVID-19 pandemic. Social distancing through partial or complete lockdowns often leads to psychological issues such as anxiety, depression, and panic attacks—all of which are known to downregulate the immune system [2, 24]. Associated economic downturns, featuring job losses and financial hardships, have accentuated mental health issues during the pandemic [25]; suicides, opioid overdoses, and domestic violence also have increased. When vulnerable persons such as children, pregnant women, or elderly relatives are part of the household, stress and anxiety levels appear to worsen, given the higher disease severity and mortality rates in these groups. The conglomeration of stress states is associated with downregulation of immunity and, consequently, with worsened disease manifestations.

### Stress

Both chronic and sub-acute stress have a significant negative impact on the immune system [26]: on the one hand, the ability to cope with stress helps preserve immune function; on the other hand, individuals with higher stress levels and poor coping mechanisms have subpar immunity. Lower resilience to stress is associated with poor antibody response and decreased natural-killer cell activity [27, 28].

Stress affects immune function by increasing glucocorticoid and catecholamine secretion. Stress also induces chronic sympathetic overdrive as it simultaneously attenuates the parasympathetic system [29]. Escalated sympathetic drive with its attendant hormonal milieu (including cortisol excess and a robust catecholaminergic drive) attenuates the efficacy of the immune system [30]. The aberrant pathophysiology at play under such conditions is increased inflammation and decreased protection against invading microorganisms [30]. Increased glucocorticoid levels significantly affect the immune function by dysregulating cytokine production, affecting natural-killer cell activity and reducing immunoglobulin A (IgA) production [30]. Elevated cortisol potentiates glucose intolerance and diabetes and thus further increases the risk for infection [31]. Moreover, evidence suggests that people who have stressful life events have greater risk for respiratory infections [32]. The higher stress levels associated with extended lockdowns and the concomitant fear, anxiety, and depression lead to weakened immunity, opening the floodgates of infection [33].

The paradoxical response of augmented inflammation that is elicited during stress despite increased corticosteroid levels in the blood is not clearly delineated. After all, chronic stressors should ameliorate the symptoms of inflammation-related diseases, but this conclusion is at odds with the excess morbidity and mortality documented in chronically stressed individuals. Miller and colleagues [34] have put forth an alternative hypothesis that posits the development of macrophage resistance to cortisol negative feedback under conditions of chronic stress, due to compensatory downregulation at the immune cell (glucocorticoid) receptor. Early life stress can give rise to blunted cortisol negative feedback of the innate inflammatory response [35]. This may set the stage for the stress-related chronic inflammation thought to lower the threshold for stress-related noncommunicable disease [36]. However, the research establishing cell surface receptor compensatory changes under conditions of stress has thus far been unimpressive. Further research is needed to discern the probable mechanism for this phenomenon.

### Depression

During lockdowns, social isolation and lack of physical activity are two prominent risk factors for depression. Depression increases the risk ofCOVID-19 infection significantly. There was increased mortality and hospitalization rates among COVID-19 infected patients having recently diagnosed depression [37].

Compared with nondepressed cohorts, individuals with recently diagnosed depression were found to have a significantly higher risk for COVID-19 infection (Adjusted Odds Ratio 7.64, 95% confidence interval 7.45- 7.83) [35, 36]. Depression is correlated with alteration in immune markers, including decreases in mitogen proliferation, natural-killer cell activity, and the types and respective quantities of antibodies produced [38]. Depression also dysregulates the neuroendocrine system [39] and consequently increases inflammation, altering the immune system’s effectiveness while simultaneously increasing bystander damage [40]. Patients with depression have disrupted T-cell function and elevated levels of cytokines, such as tumor necrosis factor (TNF)-α, IL-1, and IL-6 [40].

### Anxiety

Pandemics are associated with heightened anxiety, on both the collective and individual levels. The highly contagious nature of COVID-19 and the lack of treatment options add to the increased threat to survival and may trigger or aggravate existing anxiety and panic disorders.

Anxiety contributes to significant dysfunction in immune function by dysregulating the hypothalamic-pituitary-adrenal (HPA) axis [41, 42]. In a study of 42 patients with panic disorder and 42 healthy individuals, Koh and Lee observed significantly lower IL-2 production and lymphocyte proliferation levels in patients with anxiety disorder than in those without [43]. Complex changes in the inflammation milieu related to aberrant cytokines, particularly IL-1β, IL-6, TNF-α, and interferon (IFN)-γ, have been documented in anxiety-based disorders [44]. Furthermore, patients with anxiety disorder exhibit lower CD4+ cell counts, compared with healthy controls. Studies have also documented the elevation of suppressor CD8+ cells in these conditions, along with a potentiated cytokine response [45]. This abnormal response of the body’s immunological system in anxiety and depression may contribute to heightened infection and mishandling of severe infection, leading to a magnified, self-damaging cytokine response [46].

## Yoga and immunity

Yoga is noted to have a positive impact on the immune system [47–49] and inflammation pathways (Table 1). It reduces inflammation and increases the number and activity of natural-killer cells [50–52], thus enhancing cell-mediated cytotoxicity of invading infective agents. Evidence shows that yoga practice is associated with improvement in CD3+ and CD4+ cell counts, salivary cortisol levels, and IgA [53], a dominant player in innate immunity that is present on body linings, such as those of the lungs and the gastrointestinal tract [54]. With yogic intervention, IgA levels increase at the exposed lung border, where type II pneumocytes are prevalent. Additionally, cortisol, which dampens the body’s ability to fight infection, is decreased by practicing yoga.

**Table 1:** Studies on Yoga and Immunity

Author/Year	Sample size	Participant characteristics	Location/ Setting of study	Study design	Intervention	Results	Conclusion
Agnihotri et al., 2014 [40]	276	patients of mild to moderate asthma (FEV 1 > 60%) aged between 12 to 60 years	Department of Pulmonary Medicine, King George’s Medical University, U.P., Lucknow, India	Randomized controlled trial	6-week yoga intervention (30 minutes/day, 5 days/week of asana and pranayama)	Decreased eosinophil and neutrophil counts among patients with asthma in yoga group	Asana and pranayama help to improve hemoglobin counts and to decrease bronchial inflammation
Chen et al., 2017 [50]	94	94 healthy pregnant women at 16 weeks’ gestation	a prenatal clinic in Taipei	longitudinal, prospective, randomized controlled trial	20-week yoga intervention (60 minutes/day, twice a week of asana and pranayama)	Significantly lower cortisol levels; high IgA; improvement in CD3+ and CD4+ cell counts in yoga group	Asana and pranayama bolster immune response by reducing cortisol levels and increasing IgA and CD3/4+ counts
Naoroibam et al., 2016 [45]	44	HIV-1 infected individuals	Two HIV rehabilitation centers of Manipur State of India	A randomized controlled pilot study	1-month yoga intervention (60 minutes/day, 6 days/week of asana and pranayama)	Significantly higher CD4+ cell counts in yoga group	Asana and pranayama improve immunity in HIV-1–infected adults
Kuloor et al., 2019 [53]	60	HIV-positive (aged 30-50 years)	Rehabilitation centres across Bangalore	A randomized controlled study	8-week yoga intervention (60 minutes/day, 5 days/week of asana and pranayama)	Significantly lower rates of anxiety, stress, and depression in yoga group	Asana and pranayama help lower stress, anxiety, and depression levels of HIV-positive patients
Yadav et al., 2012 [55]	86	Patients with chronic inflammatory diseases and overweight/obese subjects	Integral Health Clinic, Department of Physiology, All India Institute of Medical Sciences, New Delhi, India.	Preliminary results from a nonrandomized prospective ongoing study with pre-post design.	10-day yoga intervention (asana and pranayama)	Decreased levels of cortisol, IL-6, and TNF-α; increased β-endorphin levels	Asana and pranayama reduce inflammation and stress levels over a short span of intervention
Rao et al., 2008 [39]	98	Recently diagnosed stage II and III breast cancer patients	Comprehensive cancer care center in Bangalore, India	Randomized controlled trial	1-month yoga intervention (pranayama)	Increased CD56+ cell counts in yoga group	Pranayama bolsters innate immunity after surgery

*IgA* denotes immunoglobulin A, *IL* interleukin, *TNF* tumor necrosis factor

Yoga has been found to be effective in immunocompromised conditions such as HIV. It helps to improve CD4+ count and anxiety, depression, and stress among patients with HIV [47, 56]. It has found to be equally effective in improving CD56+ cell count, anxiety, and depression in chronic disorders such as cancer [51].

The cytokine storm unleashed by the body’s unregulated response to SARS-CoV-2 induces multiorgan damage, resulting in high morbidity and mortality. Myocarditis with severe refractory acute heart failure has been noted [57]. As myocarditis is a clear signal for cytokine-mediated damage, direct damage by the SARS-CoV-2 virus cannot be discounted, as both the heart and vascular endothelium express the ACE2 receptors that are entry gates for COVID-19 [13]. Cytokine profiles in patients diagnosed with COVID-19 showed marked elevation of T-helper lymphocyte type 1, IFN-γ, and inflammatory cytokines IL-1β, IL-6, and IL-12 for at least 2 weeks after disease onset [58]. Among these, IL-6 is a predictor of mortality in COVID-19 patients, which may explain why primary evidence suggests that IL-6 inhibitors have shown promise as treatments [2, 59].

Nagarathna et al. have documented the downregulation of pro-inflammatory markers by yoga in their review article, hence supporting the utility of yoga as a complementary intervention for subjects at risk or already infected by SARS-CoV-2 virus [60]. Evidence indicates that yoga practice helps to reduce inflammation by downregulating a vast array of initiators and modulators that perpetuate chronic inflammation, including IL-6, TNF-α, and IL-1β [59, 60].

Multiple randomized controlled trials have documented a significant reduction in IL-6 levels in yoga groups as compared with controls [61]. In one study, researchers observed a significant reduction in IL-6 at the 3-month follow-up in breast cancer patients who practiced yoga, compared with a non-yoga control group [62]. Moreover, increasing the amount of yoga practice led to a more pronounced decrease in IL-6, pointing towards a potential dose-response effect. Another randomized trial showed significantly reduced IL-6 secretion after yoga practice in healthy individuals and significantly reduced secretion of IL-6 when cultured blood was challenged with a toll-like receptor agonist [62]. Multiple studies have substantiated the beneficial effect of yoga on inflammation and how it leads to CRS reduction, if not inhibition (Table 2).

**Table 2:** Studies on Yoga and Inflammation

Author/Year	Sample size	Participant characteristics	Location/ Setting of study	Study design	Intervention	Results	Conclusion
Kiecolt-Glaser et al., 2014 [63]	200	Breast cancer survivors	The Ohio State University, Columbus, OH.	A randomized controlled trial	12-week yoga intervention (twice weekly) among breast cancer survivors	Significant decrease in IL-6, TNF-α, and IL-1β	Yoga practice helps reduce inflammation
Chen et al., 2016 [61]	30	Healthy, female Chinese subjects	School of Public Health, Soochow University, Jiangsu Province, China	A Randomized Clinical Trial	8-week Hatha yoga intervention (twice weekly) among healthy females	Significant decrease in IL-6, IL-8, IL-1β, and TNF-α	Yoga intervention improves risk for metabolic disorder and inflammatory cytokine dysregulation
Rajbhoj et al., 2016 [64]	48	Male industrial workers	Scientific Research Department, Kaivalyadhama, Lonavla, Pune, Maharashtra, India.	A Randomized Clinical Trial	12-week yoga intervention among healthy male participants	Significant decrease in IL-10 and IL-1β	Yoga practices could reduce pro- and anti-inflammatory cytokines

*IL* denotes interleukin, *TNF* tumor necrosis factor

## Yoga during stressful events

Various clinical trials have suggested a significant role for yoga in reducing depression and its associated variables (Table 3). In one study, 16 distressed women received 3 months of Iyengar yoga intervention, and a group of 8 women served as a control. After 3 months, women in the yoga group showed a significant decrease in perceived stress, depression, and anxiety and in salivary cortisol; well-being improved significantly in the yoga group, compared with controls [65].

**Table 3:** Studies on Yoga and Stress, Anxiety and Depression

Author/Year	Sample size	Participant characteristics	Location/ Setting of study	Study design	Intervention	Results	Conclusion
West et al., 2004 [66]	69	Healthy college students	Reed College, USA	Longitudinal cohort study	90-minute Hatha yoga session	Significant reduction in titers, negative affect, and cortisol	Hatha yoga reduces both cortisol and perceived stress level
Michalsen et al., 2005 [67]	24	24 self-referred female subjects who perceived themselves as emotionally distressed	Germany	Controlled prospective non-randomized study	3-month Iyengar yoga intervention among mental distressed women	Compared to the control groups significant reduction in perceived stress was observed	Yoga helps to improve perceived stress among distressed women
Janakiramaiah et al., 2000 [68]	45	Untreated melancholic depressive patients	Department of Psychiatry, National Institute of Mental Health and Neurosciences, Bangalore, India.	Randomized comparative trial	Sudarshan Kriya for 4 weeks among patients with melancholic depression	Significant reduction in depression score	Sudarshan Kriya demonstrated its antidepressant effects in depression
Smith et al., 2007 [65]	131	Subjects with mild to moderate levels of stress	Community in South Australia	A randomised comparative trial	10-week Hatha yoga intervention	Significant improvement in SF-36 scores was observed in yoga group	Hatha yoga intervention helps to improve stress, anxiety and health status compared to relaxation
Naveen et al., 2016 [69]	54	Adult outpatients with Major Depression	Out-patient services of NIMHANS, Bangalore, India	Prospective cohort study	3-month yoga intervention among patients with depression	Significant improvement in depression, BDNF, and serum cortisol was observed	3 month yoga intervention helped improve BDNF, cortisol, and depression in depressive patients
Streeter et al., 2012 [70]	34	Normal subjects with no prior yoga experience	Community in USA	Randomized comparative trial	60-minute yoga intervention	27% increase in GABA levels in yoga group	Yoga could help a treat disorders with low GABA levels like depression, anxiety
Shelov et al., 2009 [71]	46	Normal staff and students	Ferkauf Graduate School of Psychology (FGS) and the Albert Einstein College of Medicine (AECOM) in Bronx, New York	Randomized controlled trial	8-week yoga intervention	Elevated levels of mindfulness, per Freiburg Mindfulness Inventory	Yoga increases mindfulness and potentially prevents later development of negative emotional mood states

*BDNF* denotes brain-derived neurotrophic factor, GABA γ-aminobutyric-acid

**Fig. 2:**
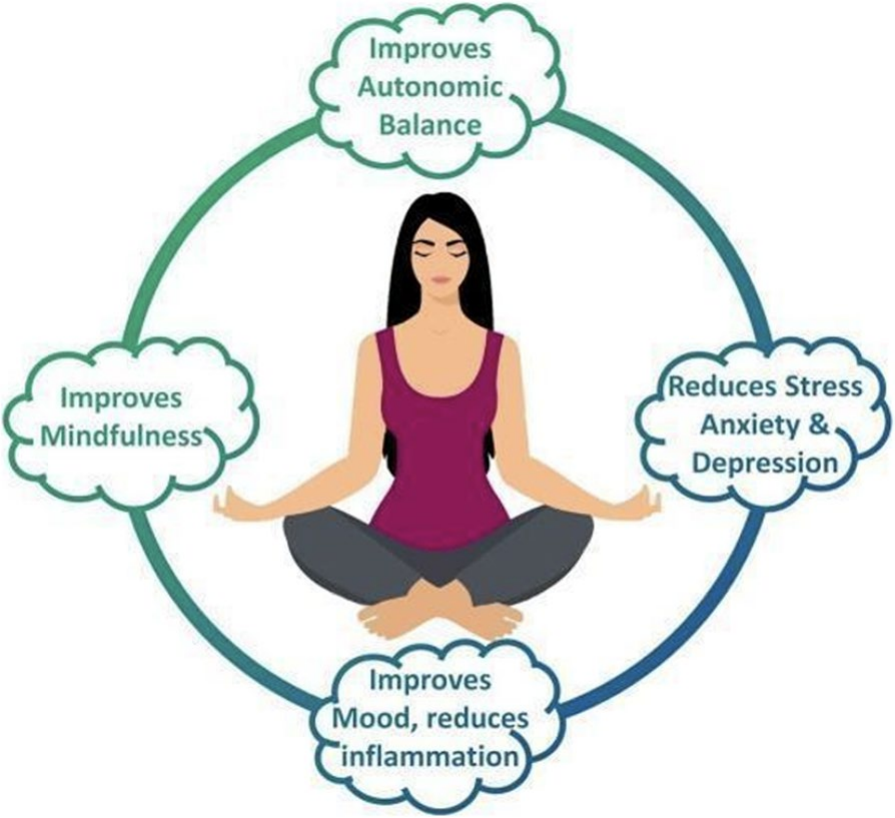
Yoga helps to improve various health parameters related to immunity. [Contribution by Mohammad A. Salem, MD; used with written permission]

Yoga practice helps adherents to develop a positive attitude during stress and to enhance self-awareness and coping ability (Fig. 2). Yoga (asana, pranayama, and meditation) improves calmness and mindfulness and increases an individual’s awareness and self-control [52]. Hatha yoga (a variation in which only yoga postures are practiced, with little or no meditation) improves HPA axis dysregulation, corrects autonomic balance, and enhances homeostasis by hastening recovery from stress [66]. In a study among 131 participants with mild to moderate stress levels, 10 weeks of a Hatha yoga intervention resulted in significant decreases in stress and anxiety, along with enhanced relaxation [70]. In another study, 90-minute Hatha yoga sessions led to a significant reduction in titers, negative affect, and cortisol levels [2, 72].

Yoga helps to reduce the allostatic load of the stress response [73]. It reduces sympathetic overactivity and improves parasympathetic tone during a stressful situation, as indicated by oxygen consumption level, heart rate, and the high-frequency component of heart rate variability [69].

In a meta-analysis by Cramer et al., yoga was found to be an effective intervention for improving depression [68]. Multiple studies have confirmed that yoga practice reduced depression and improved mood and cognitive function among patients with mild to moderate depression. This is achieved by enhancing the HPA axis function, increasing brain-derived neurotrophic factor (BDNF) levels and serotonin levels, and decreasing cortisol and inflammatory markers [68, 74, 75]. Autonomic dysfunction is a hallmark of both anxiety and depression [76]; regular yoga practice of pranayama can help improve autonomic balance by decreasing sympathetic overactivity and improving parasympathetic activity [69]. Yoga also enhances the γ-aminobutyric acid system, which is implicated in anxiety and depression [69].

Yoga also improves various cognitive facets, such as attention, concentration, memory, and executive functioning [71]. By improving body awareness, feelings, and thoughts, yoga facilitates the experience of body sensations in a nonjudgmental way [77]. It also enables the practitioner to focus on present experience instead of ruminating over future or past worries [78]. Self-awareness aids in avoiding addictive or overindulgent behaviors, including overeating and excess sleeping. Yoga helps people remain active and fosters a positive attitude during a lockdown.

## Cardio-respiratory protective effects of yoga

Given the severe cardiorespiratory illness manifested in COVID-19 [1], consistent training in yoga may play a protective role. Yoga has numerous positive effects on the cardiovascular and respiratory systems. It has been proven to improve various forms of cardiac arrhythmia, congestive cardiac failure, ischemic heart disease, and hypertension [79–83]. Regular yoga practice attenuates systolic and diastolic blood pressure and mean arterial pressure; it has also been credited with maintaining appropriate blood pressure with less medication [84]. Simply lying down in the Savasana yogic posture for 20 minutes daily was found to be effective in reducing systolic and diastolic blood pressure and the need for antihypertensive medication [85]. Yoga has been shown to improve cardiac function in patients with congestive cardiac failure [86] and to improve baroreflex sensitivity, peripheral vascular resistance, and heart rate variability [87]. It also helps to attenuate catecholamine secretion, which has been implicated in the etiology of severe cardiomyopathy and heart failure [88]. In one study, 8 weeks of yoga intervention led to significant decrease in IL-6, C-reactive protein, and extracellular superoxide dismutase, compared with non-yoga controls in patients with heart failure [89]. Thus, evidence indicates that yoga offers multi-faceted protection from cardiac damage mitigated by aberrant cytokine release, such as that seen with COVID-19.

## Limitations

Our review is up-to-date, and the findings are of significant relevance but the important limitations must be considered. The literature was searched and summarized thoroughly but our review was not systematic, thus increasing the possibilities of selection and publication bias. Our study included only articles in English thus introducing a language bias. The associations and characteristics identified in this review await clearly proven causative mechanisms. Important confounders exist in the cross-sectional studies reviewed in the form of age, medications, and immune strength. Larger randomized controlled trials will provide necessary insight on the role of yoga in immunomodulation and mental health during the present pandemic.

## Conclusions

The aggregation of pathophysiological aberrations, both psychological and somatic, secondary to COVID-19 pandemic and its resultant restrictions, may increase the severity of the infection. Accumulated evidence leads us to hypothesize that, for many, yoga practice may attenuate the ill effects of COVID-19–induced immune dysfunction at different stages.

From a public health perspective, yoga represents a low-cost, noninvasive strategy for alleviating the physical and emotional toll of the COVID-19 pandemic. The aforementioned yoga practices can be performed at home, in adherence to social distancing guidelines. Outcomes from an 8-week yoga intervention (asanas, pranayama, and meditation) indicated that medical treatment plus yoga is more effective than medical treatment alone in reducing anxiety [90]. Relaxation techniques like yoga and meditation helps in managing chronic or long term stress by regulating the cytokines, thus assisting people to overcome co-morbidities associated with diseases and improving the quality of life; which is important in COVID-19 and post-COVID illness [2, 21]. Notwithstanding, appropriate clinical trials are required to document the efficacy of this strategy.

## Data Availability

The datasets used and/or analyzed during the current study are available from the corresponding author on reasonable request.

## Abbreviations

ACE: Angiotensin-converting enzyme
BDNF: Brain-derived neurotrophic factor
COVID-19: Coronavirus disease
CRS: Cytokine release syndrome
HIV: Human immunodeficiency virus
HPA: Hypothalamic-pituitary-adrenal
IFN: Interferon
IgA: Immunoglobulin A
IL: Interleukin
TNF: Tumor necrosis factor
